# Autonomic Nervous System, Inflammation, and Diabetes: Mechanisms and Possible Interventions

**DOI:** 10.1155/2012/894157

**Published:** 2012-12-31

**Authors:** M. C. Irigoyen, Dulce Elena Casarini, Mariana Morris, Nicola Montano

**Affiliations:** ^1^Hypertension Unit, Heart Institute (InCor), University of São Paulo Medical School, Rua Eneas de Carvalho Aguiar 44, 05403-000 São Paulo, SP, Brazil; ^2^Nephrology Division, Department of Medicine, Federal University of São Paulo, 740 Botucatu Street, 04023-900 São Paulo, SP, Brazil; ^3^Department of Pharmacology & Toxicology, Boonshoft School of Medicine, Wright State University, 3640 Colonel Glenn Highway, Dayton, OH 45435, USA; ^4^Internal Medicine II, Department of Clinical Sciences, Luigi Sacco Hospital, University of Milan, Milan, Italy

It is well known that cardiac autonomic neuropathy increases morbidity and mortality and is associated with prognosis of cardiovascular events in diabetes. Indeed, autonomic imbalance between the sympathetic and parasympathetic nervous system regulation of cardiovascular function is markedly associated with mortality among patients with both type 1 and type 2 diabetes [1].

The published evidence supports a common pathogenesis for IHD, hypertension, and diabetes based on a sympathetic homeostatic shift, and the usefulness of prevention based on improving the risk/prevention balance by using standard pharmaceutical and lifestyle preventative measures [2]. 

In addition, autonomic nervous system has been indicated as an important element in the bidirectional communication between the brain and the immune system, allowing the central control of immune status and inflammation [3]. 

This special issue includes 9 papers on autonomic mechanisms, inflammation, and interventions being one of them a review. In fact, J. Petrofsky et al. examine the influence of autonomic dysfunction associated with aging and type 2 diabetes on daily life activities concentrating on how autonomic impairment alters normal daily activities. Impairments include the response of the blood vessels to heat, sweating, heat transfer, whole body heating, orthostatic intolerance, balance, and gait. In addition, the effects of ageing were examined.

In the submitted research papers, D. Senador et al. demonstrate that the effects of high-fructose diet in producing cardiovascular and metabolic pathologies depend on the timing of fructose intake, while G. Garruti and colleagues in a clinical study examine the links between metabolic syndrome and cardiovascular autonomic dysfunction. The authors suggest that metabolic syndrome not only increases the cardiovascular risk of relatively young subjects with T2D but is also associated with impaired cardiovascular autonomic function. In a very interesting research paper, D. C. Lieb et al. concluded that cardiac autonomic imbalance and adipose tissue-derived inflammation in newly diagnosed and established type 2 diabetes are interrelated. 

In the following papers, F. G. Shiraishi et al. have shown that in patients with diabetes and chronic kidney disease, aerobic capacity was associated with inflammatory state independently of diabetes presence. On the other hand, L. Jorge and colleagues demonstrate that a single bout of dynamics aerobic exercise was able to improve hemodynamic and autonomic function as expressed by baroreflex sensitivity control of heart rate in experimental diabetes. In other interventional research paper, P. Fiorino et al. examined cardiac autonomic modulation and metabolic response in streptozotocin diabetic rats treated with green tea. The authors concluded that the green tea reduced hyperglycemia and prevented renal injury and autonomic dysfunction in experimental diabetes.

Finally, S. N. Xue et al. have shown that MMP9 deters the healing of diabetic foot ulcers by inhibiting the biological behaviors of skin fibroblasts, while M. Zhang et al. have suggested that *LYR motif containing *1 gene may be an important mediator in the development of obesity-related insulin resistance since the expression of LYRM1 mRNA is affected by a variety of factors that are related to insulin sensitivity.

The purpose of the present special issue was to discuss the role of autonomic nervous system not only in cardiovascular control but also in other pathophysiological mechanisms associated with inflammation and tissue damage, believing that sympathetic and parasympathetic balance may influence the risk and prevention equilibrium in diabetes. In conclusion, restoration of autonomic balance must be the aim of the prevention programs including therapeutic lifestyle changes and other pharmacologic approaches.

*M. C. Irigoyen* *M. C. Irigoyen* *Dulce Elena Casarini* *Dulce Elena Casarini* *Mariana Morris* *Mariana Morris* *Nicola Montano* *Nicola Montano*
